# Ageing and COVID-19: What Is the Role for Elderly People?

**DOI:** 10.3390/geriatrics5020025

**Published:** 2020-04-26

**Authors:** Donatella Rita Petretto, Roberto Pili

**Affiliations:** 1 Department of Pedagogy, Philosophy and Psychology, State University of Cagliari, 09127 Cagliari, Italy; 2 Global Community on Longevity, Comunità Mondiale della Longevità, Selargius 09047, Italy; com.mond.long@gmail.com

**Keywords:** ageing, ageism, outbreak, COVID-19, active ageing, people with disability, promotion of health

## Abstract

Italy is one of the oldest countries in Europe and in the world and now it is also one of the first countries that are fighting against COVID-19. In our country, the increasing life expectancy (80.5 for males and 84.9 for females, with a total life expectancy of 82.9) has led to very positive consequences for health and the well-being of elderly people: a very high number of older adults lives and acts independently in their daily life, even if they have one or more than one chronic disease. In the time of COVID-19′s outbreak in Italy, the focus of the media was on elderly people for two main reasons. First, many older people demonstrated a very high civic sense and they were helping society to fight against the pandemic. Second, also in Italy, like in China, the older adults are at higher risk in being infected with COVID-19 and if they get ill, they have a higher risk of death. The balance previously achieved between age-related disorders and a good quality of life and good health is now under high pressure. It is very important to protect elderly people from infection, but also it is important to respect them and to support them in this complex situation. There is a great risk of “ageism”. In agreement with Lloyd-Sherlock and colleagues (2020), in this editorial we propose some hints of analysis, starting from the ongoing experience in Italy.

Italy is one the oldest countries in Europe and in the world and now it is also one of the first countries that are fighting against COVID-19. In our country, the increasing life expectancy (80.5 for males and 84.9 for females, with a total life expectancy of 82.9) [1] has led to very positive consequences for health and the well-being of elderly people: a very high number of older adults live and act independently in their daily life, even if they have one or more than one chronic disease [2,3,4]. These women and men represent a very important reference for their social, economic and cultural roles in the society. They are caregivers for grandchildren and they give economic support for unemployed younger people or with precarious jobs. Additionally, they are the historical memory of our country. Due to the increasing of life expectancy, together with the spread of healthy lifestyles, in Italy we have elderly people that seem younger than they are, and they are in very good health condition [2]. 

In the time of COVID-19′s outbreak in Italy, the focus of the media was on elderly people for two main reasons. First, many older people demonstrated a very civic sense and they were helping society to fight against the pandemic: “retired physicians and nurses” came back to work, “retired scientists and researchers” came back to work and other people helped communities in different ways (also preparing sanitary tools for protection against infection, like protective masks and disposable gowns). In the emergency, old Italian adults show a great ability to cope and help communities, even in the risk of infection for them and for their families. From previous research, we know that in Italy there are about 4 million old adults that are retired from work for age-related reasons and according to the actual laws on retirement, but they’d like to work and to have an active role in the productive system. Additionally, it is important to remember that people who are currently eighty and ninety are those who experienced the Second World War and the subsequent “economic boom”. In the past, they learned how to cope with a difficult situation and to develop a sort of a “post-traumatic growth” and now they are demonstrating it with high civic sense.

Second, also in Italy, like in China, the older adults are at higher risk in being infected with COVID-19 and if they get ill, they have a higher risk of death [5,6,7]. The higher the age, the higher the risk, with a negative effect of previous diagnosed diseases [6,8]. On March 17, 2020, the Italian National Institute of Health reported 1625 deaths (139 aged 60–69, 578 aged 70–79 and 850 age 80 and over). People 60 years old and over were about 96.5% of the total number of deaths, while in China they were about 80.8% of the total number of deaths [6]. The balance previously achieved between age-related disorders and a good quality of life and good health is now under great pressure.

Now it is very important to protect elderly people from infection, but also it is important to respect them and to support them in this complex situation. There is a great risk of “ageism”. The higher vulnerability of old adults to infection increases the risk of “ageism” and the active role that elderly people are having during outbreak reduces the risk of “ageism”.

There could be also an increasing risk that age could represent a negative factor when the acute phase of the pandemic puts high pressure on the healthcare system and the availability of resources is not enough to cope with all the needs.

With this paper, we aim to contribute to the discussion in the field of consequences of COVID-19′s outbreak, and we would like to highlight the risks of ageism, the need to put elderly people in the center of any interventions in the outbreak phase of COVID-19 and the need to personalize intervention for elderly people [4,9]. In agreement with Lloyd-Sherlock and colleagues [10], we propose some hints of analysis, starting from the ongoing experience in Italy:

- As elderly people act as caregivers of grandchildren when parents go to work, it is very difficult for them to make social isolation, home confinement and quarantine. So, we need specific interventions to support grandfathers, grandmothers and to support people who must work during pandemic [10]. In Italy, also in the complete lockdown of the country, some workers have to go to work because they work in a field related to the so-called “essential services” (like food and drugs production, public transport and other public services). So, we must guarantee them economic support.
- There is a higher risk of infection for elderly who live in rest homes, nursing homes and similar facilities. Unfortunately, these institutions can act as an incubator of infection [10,11]. Once again, the greater the age, the higher the risk and the presence of more than one disease could led them to a situation of complete dependency from the outside and from professionals. So, old adults who live in the institution need to be protected and they need to be supported with physicians and nurses. In the critical phase of the pandemic, people in institutions could have a higher risk to be isolated from the outside and they risk not receiving foods and drugs. So, we must guarantee them protection from infection and medical and social support in all the phases of the pandemic.
- In the outbreak, there is the need to share information and to spread new information in a very quick way, but elderly people could face great barriers to access information with new media, mainly due to a “digital divide” [10]. So, we must consider different kinds of mediums to spread information during the pandemic (not only “new media” but also “ancient media” like TV, a “public call” or a “municipal messenger”) and remove barriers.
- Old adults could also face great barriers to access to the health service and support, including an age-based discrimination, also for diseases and disorders different from the ones caused by COVID-19 (mental health diseases, cardiovascular diseases, tumors, neurodegenerative diseases and other age-related disorders).
- Isolation and home confinement could also increase some mental problems in elderly [12] and have very negative effects on their psychological status. Psychological support in the outbreak is necessary for them, to help them to cope with the new situation. Once again, due to the “digital divide” the diffuse use of new technological devices for social contact could be very difficult for them and other forms of social contacts in home confinement need to be studied for them (like the use of a telephone for consultations and more frequent telephone calls from relatives, friends, social workers, clinicians and volunteers) [12,13].
- Moreover, during quarantine, elderly people who live alone or who live in a difficult situation could have difficulty in having foods, drugs and other supplies [10,11,12]. A social formal and informal network is necessary during quarantine to help old adults, both in little cities and in bigger cities. In this field, the role of municipalities and social workers is central.
- Again, during quarantine, specific attention should be paid to old adults with disabilities. For them, social distance, the use of protective masks and gloves could be very disabling, because it could reduce their communication power (mainly per people with sensorial limitations). The use of protective gloves could be very disabling for people with visual disabilities, where tactile abilities are necessary to read, communicate and know the environment. The home confinement could also render complex home visits by family, friends, social workers and other professionals that could support people during daily life. So, inclusion people with a disability need to be considered in all the phases of the outbreak, with a focus on the accessibility of information and communication.

Again, in agreement with Lloyd-Sherlock and colleagues [10], we highlight the importance of international and national attention to old adults, during the outbreak of COVID-19. We believe that, even in complex situations, awareness of different aspects of a situation is the first step. Then it is necessary to take all aspects into account [14,15]. From an international point of view, the World Health Organization has produced some guidelines, with a focus on elderly people and people with disabilities [10,11]. From a national perspective, the government, scientific societies, stakeholders and health policy makers have a central role in planning and implementing a specific intervention for old adults. We believe that the second step is an approach based on planning and action: international organizations and committee, stakeholders, health policy makers and clinicians should collaborate with communities, social workers and self-advocacy associations to support old adults to overcome the outbreak. Additionally, we also highlight the importance of a long-term perspective of supporting old adults over the course of the outbreak of COVID-19 and all the future phases of the pandemic. We need to look also to the future and to schedule the progressive reduction of restrictions and of the lockdown. Additionally, we need to look further and toward the “reconstruction” after the emergency. In each phase, a person-centered and age-friendly approach is needed. Last but not least, learning from ongoing experience and the significant role played by the so-called “retired” people in facing the emergency, a new approach to age is needed. The main question is: considering the important role that old adults are playing now in the emergency, what could be their “new role” in the near future? In the next phase of “reconstruction”, what role is there for ageing people in a time where there will be a great need of the efforts of each person? From this point of view, we have a great opportunity to learn from the difficulties and to capitalize the great civic efforts of “active elderly” during the outbreak.
